# Trial of exercise to prevent HypeRtension in young adults (TEPHRA) a randomized controlled trial: study protocol

**DOI:** 10.1186/s12872-018-0944-8

**Published:** 2018-11-06

**Authors:** Wilby Williamson, Odaro J. Huckstep, Eleni Frangou, Afifah Mohamed, Cheryl Tan, Maryam Alsharqi, Mariane Bertagnolli, Winok Lapidaire, Julia Newton, Henner Hanssen, Richard McManus, Helen Dawes, Charlie Foster, Adam J. Lewandowski, Paul Leeson

**Affiliations:** 1 Oxford Cardiovascular Clinical Research Facility, Division of Cardiovascular Medicine, Radcliffe Department of Medicine, University of Oxford, Oxford, UK; 2 Centre for Statistics in Medicine, Nuffield Department of Orthopaedics, Rheumatology and Musculoskeletal Sciences, University of Oxford, Oxford, UK; 3 Centre Integré Universitaire de Santé et de Services Sociaux du Nord-de-l’Île-de-Montréal, Hôpital du Sacré-Cœur de Montréal Research Center, Montréal, Canada; 4 Nuffield Department of Orthopaedics, Rheumatology and Musculoskeletal Sciences, University of Oxford, Oxford, UK; 5 Department of Sport, Exercise and Health, University of Basel, Basel, Switzerland; 6 Nuffield Department of Primary Health Care Sciences, University of Oxford, Oxford, UK; 7 Faculty of Health and Life Sciences, Oxford Brookes University, Oxford, UK; 8 School of Policy Studies, University of Bristol, Bristol, UK; 9 Oxford Centre for Clinical Magnetic Resonance Research, Division of Cardiovascular Medicine, Radcliffe Department of Medicine, University of Oxford, Oxford, UK; 10 Oxford Cardiovascular Clinical Research Facility, Division of Cardiovascular Medicine, Radcliffe Department of Medicine, University of Oxford, John Radcliffe Hospital, Oxford, OX3 9DU UK

**Keywords:** Young adult, Blood pressure, Hypertension, Prehypertension, Preterm birth, Randomised trial, Exercise, Cardiac imaging, Cardiac Remodelling, Cerebrovascular health

## Abstract

**Background:**

Hypertension prevalence in young adults has increased and is associated with increased incidence of cerebrovascular and cardiovascular events in middle age. However, there is significant debate regards how to effectively manage young adult hypertension with recommendation to target lifestyle intervention. Surprisingly, no trials have investigated whether lifestyle advice developed for blood pressure control in older adults is effective in these younger populations.

**Methods/Design:**

TEPHRA is an open label, parallel arm, randomised controlled trial in young adults with high normal and elevated blood pressure. The study will compare a supervised physical activity intervention consisting of 16 weeks structured exercise, physical activity self-monitoring and motivational coaching with a control group receiving usual care/minimal intervention. Two hundred young adults aged 18–35 years, including a subgroup of preterm born participants will be recruited through open recruitment and direct invitation. Participants will be randomised in a ratio of 1:1 to either the exercise intervention group or control group. Primary outcome will be ambulatory blood pressure monitoring at 16 weeks with measure of sustained effect at 12 months. Study measures include multimodal cardiovascular assessments; peripheral vascular measures, blood sampling, microvascular assessment, echocardiography, objective physical activity monitoring and a subgroup will complete multi-organ magnetic resonance imaging.

**Discussion:**

The results of this trial will deliver a novel, randomised control trial that reports the effect of physical activity intervention on blood pressure integrated with detailed cardiovascular phenotyping in young adults. The results will support the development of future research and expand the evidence-based management of blood pressure in young adult populations.

**Trial Registration:**

Clinicaltrials.gov registration number NCT02723552, registered on 30 March, 2016.

## Background

Hypertension contributes to 9 million annual global deaths [1] and more disability-adjusted life years (DALYs) lost than any other single cause [2]. Lifetime risk of cardiovascular events is associated with modest increases in systolic blood pressure above 115 mmHg [2]. Acknowledgement of these risks have prompted a recent review of international criteria for hypertension diagnosis, with several proposing stage 1 diagnosis be lowered from the threshold of 140 mmHg systolic or 90 mmHg diastolic down to 130 mmHg systolic or 80 mmHg diastolic [3]. Across the life course this diagnostic change has greatest significance for adults aged 18 to 39, in whom an estimated 30–40% will now reach diagnostic criteria for hypertension leading to a 3 fold increase in prevalence [4] [5]. Such a change could help tackle a global trend of increased incidence of stroke and cardiovascular events in young adults [6, 7]. However, there is significant debate regards how to achieve lower target blood pressures in young adults [8, 9]. Some trials in middle age adults at intermediate risk have identified no benefit associated with antihypertensive therapy and potential harm in pharmacological reduction of high normal blood pressure [10].

Globally, modifiable risk factors have the largest attributable risk to cardiovascular events and stroke [11] and current guidelines recommend targeted lifestyle intervention, and in particular physical activity promotion, for young adults with hypertension [12]. However, our recent systematic review of exercise intervention trials for blood pressure identified bias in recruitment towards older adults, lack of investigation of determinates of physiological and behavioural exercise remodelling and limited understanding of the cardiovascular adaptations associated with change in modifiable risk profiles [13]. Therefore, there is limited trial evidence to support physical activity intervention as a primary treatment option at present [14]. In addition, at younger ages, a range of factors including birth history, familial predisposition and young adult socio-economic exposures have influences on cardiac and cerebrovascular remodelling that may make cardiovascular response to lifestyle interventions more complex [5, 15, 16]. This means there is lack of understanding regards effectiveness of intervention across subgroups of the population and potential heterogeneity in response to exercise training [15–22]. Therefore, this study will trial an exercise intervention in a young adult population with high and high normal blood pressure to identify effectiveness for management of blood pressure and also understand determinates of behavioural and physiological remodelling as well as differential effects across phenotypes.

### Study aims and objectives

The primary aim of this study is to compare the effect of a structured aerobic exercise and physical activity intervention versus usual care/minimal intervention on ambulatory blood pressure levels in young adults with high normal or elevated blood pressure. Secondary aims include investigation of the broader effects of exercise on peripheral and central cardiovascular remodelling, metabolic function, physical activity behaviours, and physical function. Tertiary aims target an exploratory analysis of remodelling across the liver, heart and brain using MRI indices of structure and function and will explore the potential that baseline phenotypes predict exercise response (Table 1**).**

**Table 1:** TEPHRA trial objectives, outcome measures and measurement time-points

Objectives	Measures	Time-points
Benefits of Exercise		
• Short term Blood Pressure Response: The primary objective is to compare the effect of aerobic exercise intervention and physical activity self-monitoring versus usual care/minimal intervention on ambulatory blood pressure levels in young adults with high normal and elevated blood pressure.	– Systolic and diastolic blood pressure measured during ambulatory blood pressure monitoring	Measured at baseline and at 16 week follow-up
• Sustained blood pressure response: To compare the sustained effect of aerobic exercise intervention with continued physical activity self-monitoring and motivational coaching versus usual care/minimal intervention on awake blood pressure levels in young adults with high normal and elevated blood pressure.	– Systolic and diastolic blood pressure measured during ambulatory blood pressure monitoring	– baseline – 16 weeks follow-up – 52 weeks follow-up
• Cardiovascular Fitness: To compare the effect of aerobic exercise intervention and physical activity self-monitoring versus usual care/minimal intervention on cardiovascular performance and stress response in young adults with high normal and elevated blood pressure.	– Cardiopulmonary exercise testing - Oxygen uptake and carbon dioxide exchange kinetics across submaximal and peak exercise – Dynamic central and peripheral cardiovascular response to exercise stress – Circulating plasma stress biomarkers collected pre and post exercise. – Haemodynamic response to exercise: blood pressure, heart rate, measures of cardiac performance during cardiopulmonary exercise testing. – Exercise stress echocardiography (subgroup)	– baseline – 16 weeks follow-up – 52 weeks follow-up
• Physical Function: Compare the 16-week and sustained effect of intervention on objectively measured walking gait, walking cadence and global physical function.	– Objective measure of physical activity (7 day wear of activity monitor) – Objective gait analysis – Questionnaire	– baseline – 16 weeks follow-up – 52 weeks follow-up
• Cardiac Remodelling: Investigate cardiac adaptation and remodelling following intervention, with reference to gestational age and baseline cardiovascular phenotypes.	Cardiac echocardiography measures Cardiac MRI measures (subgroup) – Cardiac mass – Left and right ventricular structure and function – 3D-shape and functional analysis	– baseline – 16 weeks follow-up – 52 weeks follow-up (echocardiography only)
• Cerebrovascular Remodelling: Investigate cerebrovascular adaptation and remodelling following intervention, with reference to gestational age and baseline cerebrovascular phenotypes.	Brain MRI (subgroup) – White and grey matter volumes – Subcortical nuclei volumes – Cortical thickness – White matter integrity – White matter hyperintensities – White matter connectivity – Brain vessel morphology – Brain vascular resistance – Brain blood flow and arrival time	– baseline – 16 week follow-up
• Hepatic Remodelling: To investigate hepatic remodelling and mean change in hepatic adiposity pre and post exercise intervention compared to control.	Liver MRI (subgroup) – Structure and volume – Intra-hepatic lipid content – Steatohepatitis – Hepatic fibrosis – Hepatic Iron Load	– baseline – 16 weeks follow-up
• Metabolic Function: Compare the effects of intervention on the fasting metabolic profile.	– Fasting glucose profile – Fasting insulin profile – Fasting lipid profile	– baseline – 16 weeks follow-up – 52 weeks follow-up
• Retinal and Dermal microvascular structure: Compare the effect of intervention on retinal and dermal vascular structures.	– Retinal imaging arteriolar and venular indices – Dermal capillary density	– baseline – 16 weeks follow-up – 52 weeks follow-up
Mediators of Exercise Adaptation and Blood Pressure Response		
• Physical activity behaviours: Compare the 16-week and sustained effect of intervention on objectively measured ambulatory physical activity and sedentary behaviour in young adults with high normal and elevated blood pressure.	– Objective measure of physical activity (7 day wear of activity monitor)	– baseline – 16 weeks follow-up – 52 weeks follow-up
• Molecular endothelial and angiogenic function: Compare the effect of aerobic exercise intervention versus usual care/minimal intervention on circulatory markers of angiogenesis and endothelial colony-forming cells (ECFC) function and association with change in blood pressure and cardiovascular fitness.	– Circulatory markers of angiogenic function. – ECFC colony growth (before or after 15 days in culture) – ECFC function: ECFC proliferation rate and number of branches and closed tubes formed on matrigel – Systolic and diastolic blood pressure measured during ambulatory blood pressure monitoring – Cardiopulmonary exercise testing	– baseline – 16 weeks follow-up
• Vascular remodelling: Compare the effect of aerobic exercise intervention and physical activity self-monitoring versus usual care/minimal intervention on central blood pressures, vascular stiffness and vascular structures.	– Pulse wave velocity – Augmentation index – Central blood pressure – Retinal imaging arteriolar and venular indices – Dermal capillary density	– baseline – 16 weeks follow-up – 52 weeks follow-up
Predictors of Response to Exercise and Blood Pressure Change		
• Perceptions of the study and intervention compliance: Track compliance with the intervention and characterize participants’ subjective and qualitative experience of intervention and correlate with intervention effects on blood pressure and cardiovascular fitness to assess efficacy of the intervention.	– Exercise log and Fitbit step counts via Fitabase – Structured interview of participants	– 0–16 weeks training log – 16–52 weeks step count – End of trial period
• Physical activity beliefs: Investigate the correlation between physical activity behaviour change and participants’ cognitive and psycho-social determinants of exercise including self-efficacy to exercise, motivations to exercise.	– Objective measure of physical activity (7 day wear of activity monitor) – Self-reported questionnaire responses, including self-reported physical activity questionnaires, cognitive and psycho-social questionnaire items and self-efficacy measures.	– baseline – 16 weeks follow-up – 52 weeks follow-up
• Baseline cardiovascular phenotypes: Investigate the associations between baseline cardiovascular phenotypes including the preterm born phenotype and response to exercise intervention across outcomes.	– Systolic and diastolic blood pressure measured during ambulatory blood pressure monitoring – Cardiopulmonary exercise testing - Oxygen uptake and carbon dioxide exchange kinetics across submaximal and peak exercise – Cardiac remodelling: echocardiography and cardiac MRI	– baseline – 16 weeks follow-up – 52 weeks follow-up
• Baseline cerebrovascular phenotypes: Investigate the associations between baseline cerebrovascular structures; white matter connectome, subcortical volumes (caudate, thalamus and hippocampus) and cortical thickness (across insular, precuneus and posterior cingualate) and responsiveness to exercise intervention across outcomes.	– Systolic and diastolic blood pressure measured during ambulatory blood pressure monitoring – Cardiopulmonary exercise testing - Oxygen uptake and carbon dioxide exchange kinetics across submaximal and peak exercise – Cardiac remodelling echocardiography and cardiac MRI	– baseline – 16 weeks follow-up – 52 weeks follow-up
• Tertiles of blood pressure and cardiovascular risk: Investigate differences in response to exercise intervention across outcomes in associations with baseline tertiles of the study population blood pressure and cardiovascular risk scores.	– Systolic and diastolic blood pressure measured during ambulatory blood pressure monitoring – Cardiopulmonary exercise testing - Oxygen uptake and carbon dioxide exchange kinetics across submaximal and peak exercise – Cardiac remodelling echocardiography and cardiac MRI	– baseline – 16 weeks follow-up – 52 weeks follow-up
• ECFC function: Investigate if baseline molecular and cellular mechanisms in ECFC predict response to exercise intervention across outcomes.	– ECFC proteomics – ECFC molecular and cellular responses to in vitro shear and metabolic stresses. – Systolic and diastolic blood pressure measured during ambulatory blood pressure – Oxygen uptake and carbon dioxide exchange kinetics across submaximal and peak exercise – Whole, plasma, and serum blood samples at rest. – Retinal imaging arteriolar	– baseline – 16 weeks follow-up

### Hypotheses

The primary hypothesis is that structured aerobic exercise training and physical activity self-monitoring will improve ambulatory blood pressure measures for young adults with elevated blood pressure. The secondary hypotheses are that: structured aerobic exercise training and physical activity self-monitoring will also improve other cardiovascular, metabolic and cerebrovascular risk profiles in young adults; that the degree of improvement will associate with changes in vascular biology and behaviour, as potential mediators of the changes; and thirdly that response to exercise will vary depending on baseline phenotypic differences between individuals.

## Methods

**Fig. 1:**
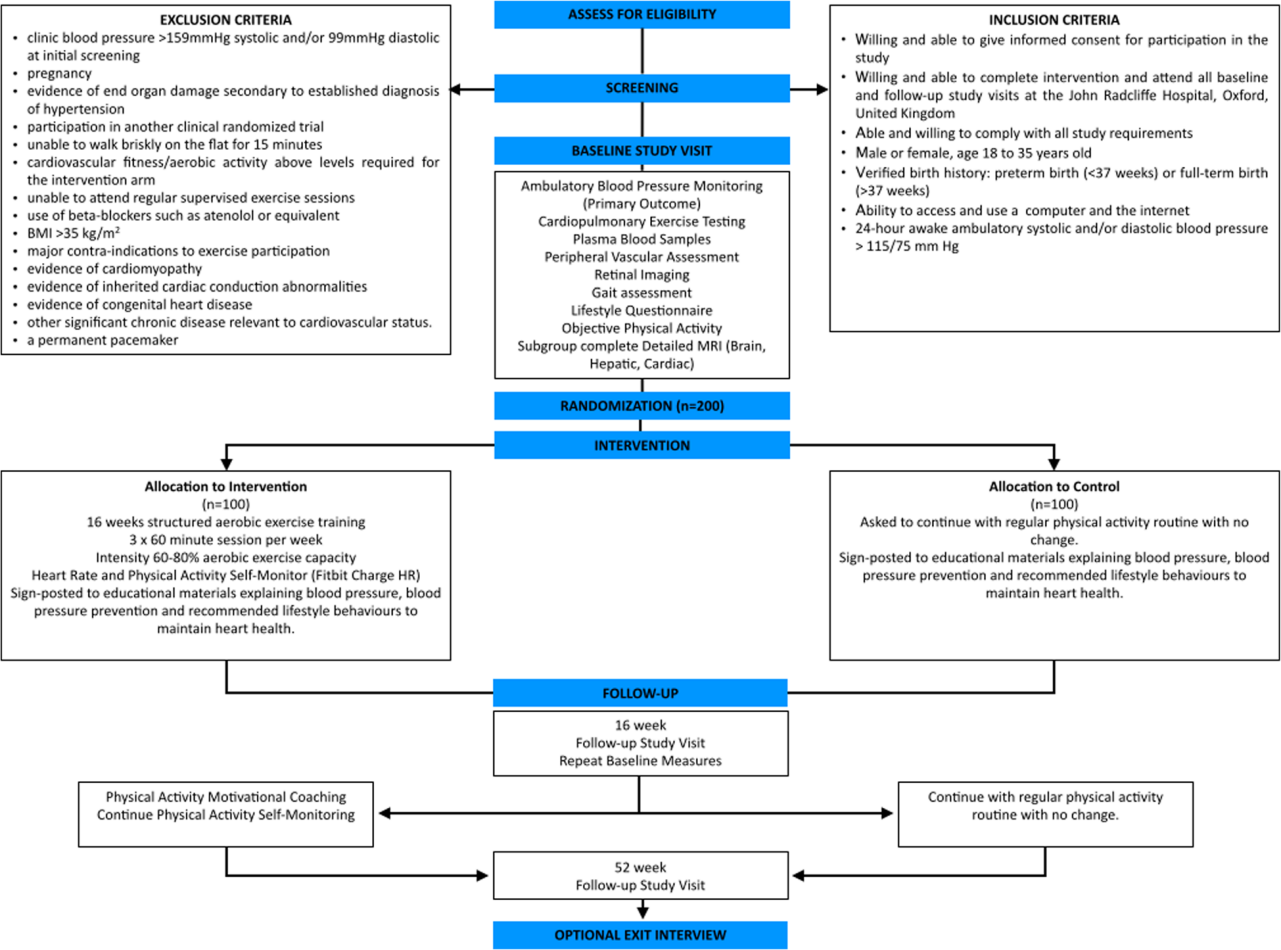
TEPHRA Trial Overview and Visit Schedule. Provides an overview of the study, describing the study inclusion and exclusion criteria, study visits, and summary of intervention arms

TEPHRA is an open label, prospective single-blinded, two-arm, parallel, randomized controlled trial in young adults with elevated blood pressure. In total, 200 participants will be randomized (ratio 1:1) following baseline measures to either exercise intervention or to a comparison control group with minimal intervention. There are two follow-up visits post-randomisation: at 16 weeks and 52 weeks (Fig. 1). The primary endpoint will be at 16 weeks post-randomisation, immediately after the end of the intervention. The protocol is prepared in accordance with the SPIRIT statement.

Participants who enter the trial will nominally complete 4 study visits over the course of the trial:

I. Screening assessment (Visit 0)
II. Baseline Study Visit (Visit 1)
III. 16-week follow-up Study Visit (Visit 2) at 16 weeks (post completion of structured exercise intervention for those in the intervention arm)
IV. 52-week follow-up Study Visit (Visit 3) - (52 weeks post randomisation)
  Study measure collection is reduced for Visit 3 to reduce redundant data collection of selected non-primary outcome data and minimise excess burden on study participants.

### Eligibility and recruitment

Recruitment strategies will include invitation from general practice (GP) records, invitation from hospital birth registers, open recruitment, targeted online recruitment, and invitation following participation in previous studies. Participants will be verified as preterm (born before 37 weeks gestation) or full-term (born after 37 weeks gestation).

### Eligibility criteria

The trial inclusion criteria are listed below.

Eligible participants will:

- be willing and able to give informed consent for participation in the study
- be willing and able to complete intervention and attend all baseline and follow-up study visits at the John Radcliffe Hospital, Oxford, United Kingdom
- be able (in the investigator’s opinion) and willing to comply with all study requirements
- be male or female, from 18 to 35 years old
- have verified birth history: preterm birth (< 37 weeks) or full-term birth (> 37 weeks)
- have the ability to access and use a computer and the internet
- have 24-h awake ambulatory systolic and/or diastolic blood pressure > 115/75 mmHg

The participant may not enter the study if any of the following apply:

- clinic blood pressure > 159 mmHg systolic and/or 99 mmHg diastolic at initial screening
- pregnancy
- evidence of end organ damage secondary to established diagnosis of hypertension
- simultaneous participation in another human or clinical randomized trial (if there is any possibility of compromising health, safety, or well-being, or any possible compromise of study data)
- unable to walk briskly on the flat for 15 min
- those currently maintaining levels of cardiovascular fitness and aerobic activity at or above the levels required for the intervention arm
- unable to attend regular supervised exercise sessions
- use of beta-blockers such as atenolol or equivalent
- BMI > 35 kg/m^2^
- major contra-indications to exercise participation
- evidence of cardiomyopathy
- evidence of inherited cardiac conduction abnormalities
- evidence of congenital heart disease
- other significant chronic disease relevant to cardiovascular status
- a permanent pacemaker

The following are exclusion criteria for the MRI sub-study only:

- shrapnel injuries
- metal clips in blood vessels of the brain
- other metal or electronic implants adversely affected by the magnetic field
- an injury to the eye involving fragments of metal
- unsuitable for MRI based on responses on the MRI safety screening form.

### Setting and participants

Participants will complete a secure, online questionnaire requesting the following information:

- Sex, age at entry to the study, birth history
- Reported pregnancy status
- Medical history to identify exclusionary medical conditions or pharmaceutical usage
- Self-reported current physical activity levels
- Willingness to change physical activity and ability to access and attend supervised exercise sessions for 16 continuous weeks

A trained clinical research investigator will review questionnaire responses and eligibility for study participation. The study investigator will clarify if there are significant contraindications, diseases or disorders, which in the opinion of the investigator might influence the individual’s ability to participate in the study. Potentially eligible participants will then be invited to attend a screening visit completed at the Oxford Cardiovascular Clinical Research Facility, University of Oxford.

### Informed consent

Individuals willing to participate in the study will be provided with the full participant information leaflets detailing the study information in advance of the informed consent process. Only after participants have had a minimum of 24 h to review the study information will they be invited to schedule a time for their screening visit. The informed consent process will be completed on the day of the screening study visit. The participant must personally sign and date the latest approved version of the informed consent form before any study specific procedures are performed. Written and verbal versions of the participant information leaflet and informed consent form will be presented to the participants detailing no less than: the exact nature of the study; what it will involve for the participant; the implications and constraints of the protocol; the known side effects and any risks involved in taking part. It will be clearly stated that the participant is free to withdraw from the study at any time for any reason without prejudice to future care, and with no obligation to give the reason for withdrawal. Written informed consent will be obtained by study team members who are suitably qualified and experienced, and have been authorized to do so by the principal investigator. A copy of the Informed Consent form will be given to the participant. The original signed form will be retained at the study site.

Candidates will be asked for their consent to access their medical records including birth and neonatal records. In absence of obstetric records, self-reported birth history will be used. As appropriate, participants will be asked to confirm their birth and pregnancy details with appropriate family member(s).

### Screening assessments

Anthropometric measurements including body composition measures, height, weight, body mass index (BMI), waist and hip circumference, and resting blood pressure shall be collected. Participants will have their blood pressure checked after 5 min rest using the automated mode of a validated sphygmomanometer (Dinamap V100, GE Healthcare, Chalfont St. Giles, United Kingdom). Three blood pressure readings will be taken at intervals of 1 min. For the outcome measure of clinic blood pressure at screening, the mean of the second and third readings will be used. Screening blood pressure will be assessed based on National Institute for Health and Care Excellence (NICE) guidelines [23] and are presented in Table 2**.**

**Table 2:** Responses to clinic blood pressure at screening

CLINIC BLOOD PRESSURE AT SCREENING	RESPONSE
NORMAL	
SBP < 110 & DBP < 70	Complete screening & exclude from trial
POSSIBLE PRE-HYPERTENSION	
110 ≤ SBP ≤ 139 &/or 70 ≤ DBP ≤ 89	Complete screening & conduct 24-h ABP monitoring as appropriate
POSSIBLE STAGE I HYPERTENSION	
140 ≤ SBP ≤ 159 &/or ≤ 90 DBP ≤ 99	In all cases: check for signs of end organ damage (resting 12 lead ECG, urinary spot analysis, retinal imaging) if signs of end organ damage exist, exclude from trial and refer for further assessment As appropriate: -complete screening & exclude from trial -complete screening & refer for further assessment -complete screening & conduct 24-h blood pressure monitoring Note: If subsequent 24-h awake ABP > 135/85, review case and consider referral for further assessment
POSSIBLE STAGE II HYPERTENSION	
SBP > 159	Exclude from trial & refer for further assessment
DBP > 99	Exclude from trial & refer for further assessment

Electrocardiogram (ECG) - A 12 lead ECG will be conducted and reviewed by a trained clinical research investigator. If the investigator determines that ECG results potentially shows evidence of exclusionary cardiac disease, the ECG (and candidate if appropriate) will be referred for further clinical review.

24 h Ambulatory Blood Pressure (ABP) - 24-h ambulatory blood pressure monitoring will be initiated at the end of the screening assessment session using validated, automated oscillometric, ambulatory devices (TM-2430, A&D Instruments or equivalent). Correct cuff size will be chosen based on arm circumference. Subjects will be instructed to remain still during measurements. Measurements will be automatically taken every 30 min during daytime and then hourly from 11:00 PM to 7:00 AM. Subjects will complete a diary documenting hours asleep and awake. ABP data will be verified by a trained study investigator prior to randomisation.

### Study visits assessments and study measures for visits 1–3

#### Participant preparation (all visits)

Participants will be asked to attend the Oxford Cardiovascular Clinical Research Facility, having consumed only water for 4 h beforehand.

#### Lifestyle and physical activity questionnaire (visits 1 & 2)

Study visit participants will be asked to complete the study questionnaire. The questionnaire combines validated questions piloted or used in previous studies, including question on smoking frequency,

alcohol consumption, physical activity (Recreational Physical Activity Questionnaire (RPAQ)), and self-reported determinants of physical activity including environmental perceptions, physical activity beliefs, and health related quality of life.

#### Physical examination (visits 1–3)

Assessments including body composition measures, height, weight, BMI, waist to hip ratio, gait analysis, and resting blood pressure will be taken. Participants will undergo a brief gait analysis by completing a monitored 10 m walk while wearing an accelerometer fastened to their lower back. Participants will have their clinic blood pressure measured in the same manner performed and recorded during screening.

#### Vascular measures (visits 1–3)

Resting measures of brachial-femoral pulse wave velocity will be measured using sphygmomanometer-derived indices (Vicorder, Skidmore Medical, Taunton, UK) with cuffs placed around brachial and femoral arteries to identify pulse arrival times [24]. In addition, sphygmomanometer-derived indices of aortic blood pressure will be derived from brachial blood pressure measurements (Vicorder, Skidmore Medical, Taunton, UK) [25].

### Microvascular assessments (visits 1–2)

#### Dermal capillary density

Microvascular imaging of dermal capillary beds will be done using intravital video capillaroscopy with a Leica Stereo Microscope at × 200 magnification and Schott light-emitting diode light (wavelength 450 to 610 nm, consistent with haemoglobin absorption spectrum). Imaging will be done on the dorsal surface of the middle phalanx of the left hand in 6 adjacent image fields at baseline for 1 min each. After completion of the baseline images, a small blood pressure cuff around the proximal phalanx will be inflated to 50 mmHg for 5 min, causing venous occlusion, and then the same 6 images as for the baseline will be recorded. Images will be captured using a Moticam 580 digital camera (Motic, Wetzlar, Germany), which will then stored for offline post-processing using commercially available Image ProPlus software as previously described [16].

#### Retinal vessel imaging

Static retinal vessel analysis will be performed using the Static Retinal Vessel Analyzer (SVA-T, Imedos Systems UG, Jena, Germany). For static analysis, three valid images will be taken from the retina of the left and right eye, with the optic disk in the centre [26]. Retinal arterioles and venules, coursing through an area of 0.5–1 disc diameter from the margin of the optic disc, will be identified using special analysing software identifying retinal vessels in ring-zones (Vesselmap 2, Visualis, Imedos Systems UG). Diameters will be calculated to central retinal arteriolar and venular equivalents (CRAE, CRVE), using the Parr-Hubbard formula described elsewhere. The CRAE and CRVE will be used to calculate the arteriolar-to-venular-ratio (AVR), taking the mean of the right and left eye results [26].

#### Echocardiogram (visits 1–2, optional on visit 3)

Cardiac ultrasound imaging will be used to evaluate cardiac structure and function. Cardiac ultrasound will be completed by an operator trained in echocardiography. Resting transthoracic echocardiography will be performed in the left lateral decubitus position using a commercially available Philips iE33, Philips EPIQ 7C, or equivalent cardiology ultrasound machine. British Society of Echocardiography guidelines will be followed for collection of a standard clinical imaging dataset [27].

#### Blood sampling (visits 1–3)

A fasting, venous blood sample (approximately 50 ml) will be taken at rest. For visits 1 and 2, a further venous blood sample (approximately 100 ml total) will be taken after exercise testing. Blood samples will be centrifuged, separated within 30 min, and stored at − 80 °C. Fasting lipid and metabolic profiles, including C-reactive protein, will be measured at the Oxford Hospital Biochemistry using routine clinical quality validated assays. Additional circulating biomarkers will be quantified using standard commercially available assays [16]. Circulating endothelial colony forming cells (ECFCs) and peripheral blood mononuclear cells (PMBCs) will be isolated and cultured from peripheral blood as previously described [28]. Cells in culture will be followed from days 7–30 to assess ECFC colony formation. ECFCs will then be expanded to allow cell phenotype, clonogenic and functional assessments including cell proliferation (EdU incorporation) using the Click-iTTM EdU Alexa FluorTM 488 Imaging kit (Thermo Fisher Scientific), cell migration and vascular cord formation capacity assessed by the numbers of closed tubes and branching formed on Matrigel (Corning Matrigel Matrix). Remaining PBMCs and ECFCs will be frozen in 10% dimethyl sulfoxide (Sigma Aldrich) fetal bovine serum (Gibco, Thermo Fisher Scientific) and stored in liquid nitrogen.

#### Cardiopulmonary exercise testing (CPET) (visits 1–3)

Participants will perform resting spirometry testing prior to performing a peak CPET on a seated stationary cycle ergometer (Ergoline GmbH, Germany) using a validated incremental protocol with respiratory gases collected and measured (Metalyzer 3B, Cortex Biophysik, Germany). Heart rate will be recorded using continuous electrocardiogram monitoring, rate of perceived exertion will be recorded every two minutes and blood pressure will be recorded every four minutes using a manual mercury sphygmomanometer, with dynamic echocardiography imaging collected in a subgroup. After one quiescent minute of resting measurements, participants will be instructed to maintain a rate of 60 rpm during the active portion of test, which begins with a two-minute warm-up with a 20-watt workload. After the warm-up period, workload is increased to 35 watts. To normalise test duration to approximately 8–12 min, participants who report higher activity or fitness levels will have their workload increased to 75 watts after the warm-up period. Workload will automatically increment by 15 watts each minute and participants will cycle continuously until safety termination criteria are met or exhaustion prevents them from maintaining at least 50 rpm. Participants will then complete a two-minute cool down period at 35 watts and the revolutions per minute of their preference. Following the cool down period, participants will be transferred to a reclined treatment bed for post-exercise venous blood sampling.

#### 24-h ambulatory blood pressure (visits 1–3)

Performed as described in screening measures.

#### 7 day accelerometer (visits 1–3)

This will be attached to the participant at the end of the study visit. It consists of a wrist worn accelerometer (Axivity AX3) similar in design to a wrist worn watch. Wrist worn accelerometers have high compliance and reliability and are validated measures of physical activity. Participants will be asked to wear the accelerometer for 7 days. Stamped addressed envelopes will be provided for the return of monitoring devices.

#### MRI substudy (visits 1–2) (image acquisition and post-processing)

Multi-organ MRI of the brain, liver and heart will be completed on a subgroup of study participants with images analysed for structure, function, and tissue property. Participants will be invited to join the MRI substudy from the start of recruitment until 100 participants are recruited. Thereafter, all preterm participants will be invited to complete the optional MRI protocol. Participants will undergo multimodality MRI scanning on the same Siemens 3.0 T scanner (Siemens, Munich, Germany) at the Oxford Centre of Clinical Magnetic Resonance Research, John Radcliffe Hospital, University of Oxford, United Kingdom. Participant will have fasted for at least 4 h prior to attending for the MRI measures, having only consumed water beforehand. MRI imaging will be completed prior to the exercise and other cardiovascular measures. All study measures were completed within 48 h.

#### Brain MRI

The Brain MRI protocol will include T1-weighted structural imaging (TR/TE = 2040/4.7 ms, flip angle 8°, FOV 200 mm, voxel size 1.0 mm isotropic), T2-weighted FLAIR (TR/TE = 9000/90 ms, flip angle 150°, FOV 220 mm voxel size 1.1 × 0.9 × 3.0 mm), Diffusion Weighted Imaging (DWI) (TR/TE = 8900/95 ms, 2.0 mm isotropic resolution, multiband echo-planar imaging (EPI), 64 slices, 64 diffusion weighted directions, FOV 192 mm, b-value 1500s/mm^2^, five non-diffusion weighted images, b-value 0 s/mm^2,^ with one b0 volume acquired in the reverse phase encoded direction), Time-of-Flight (TOF) MRA (TR/TE = 23/8 ms, flip angle 10°, FOV 300 mm voxel size 1.6 × 1.2 × 5.0 mm) and multi-delay vessel-encoded pseudocontinuous Arterial Spin Labelling (ASL), identical to a previously published protocol [29].

Global Cerebral Volumes and Cortical Thickness - T1-weighted structural images will be processed using the suite of FSL anat pipeline [30]. Cortical parcellation, cortical surface reconstruction, and cortical thickness estimation will be completed with Freesurfer (http://surfer.nmr.mgh.harvard.edu/).

White Matter Integrity - DWI will be pre-processed with FSL’s topup and eddy. A tensor model will be applied with DTIFit [30–33]. MRTrix single-shell constrained spherical deconvolution (CSD) [34] will be used for whole-brain tractography with 10 million streamlines, followed by SIFT streamline filtering. The resulting SIFT streamlines will be used to create binary and SIFT-weighted connectomes [34].

Subcortical Volumes - Subcortical nuclei segmentation will be completed using FSL MIST (Multimodal Image Segmentation Tool (MIST)) a fully automated segmentation tool. MIST takes advantage of unique MRI contrast properties of the subcortical nuclei to optimise shape and boundary recognition across T1, T2 FLAIR and DTI FA images [35].

Brain vessel morphology - Brain vessel segmentation will be completed on TOF MRA imaging using previously described automated segmentation tools [36, 37]. The binary segmentations will be used to determine global and regional brain vessel morphology (vessel density, caliber and tortuosity). All vessel segmentation will be visually checked to ensure proper quality. Vessel tortuosity will be defined by the deviation from the shortest path between two points. This analysis will be implemented by identifying the vessel endpoints and bifurcations, calculating the shortest path and the length of the actual centerline between each two connected points. The final tortuosity will then be calculated by the ratio and it will be averaged over all vessel segments.

Cerebral perfusion - Brain blood flow and blood arrival time will be estimated from ASL images using a previously described analysis pipeline and FSL BASIL [29, 38].

White matter hyperintensity (WMH) lesions – WHM will be automatically segmented on FLAIR images with BIANCA (Brain Intensity AbNormality Classification Algorithm) a fully-automated, supervised method for WMH detection [39, 40]. BIANCA classifies the image’s voxels based on their intensity and spatial features, where the intensity features were extracted from T2-weighted FLAIR, T1-weighted and DTI fractional anisotropy (FA) images. WMH masks will be manually segmented from 10 images to use as the training set for BIANCA, these will be independently verified. BIANCA probability output maps will be visually checked for quality. Lesion count and volume will be calculated, lesion count has previously been described as a measure of burden of white matter hyperintensities, however global and regional volume of white matter hypertensities has been reported as sensitive measure to track in trial settings. The minimum lesion size used in analysis will be 1 mm^3^. T1-weighted structural images will be processed using the suite of FSL analysis tools [30].

#### Cardiac MRI

Steady-state free-precession cine sequences will be used to acquire localization images, followed by optimized left ventricular horizontal and vertical long-axis cines. From these, a left ventricular short-axis cine stack will be obtained with standardized basal slice alignment with a 8-mm slice thickness and 2-mm interslice gap. All cardiovascular magnetic resonance imaging will be prospectively ECG gated with a precordial 3-lead ECG and will be acquired during end-expiration breath holding. Image acquisition parameters for the steady-state free-precession images are as follows: echo time, 1.5 milliseconds; repetition time, 3.0 milliseconds; and flip angle, 60°. The short- and long-axis steady-state free-precession images will be stored on a digital archive for post processing and analysis.

Quantification of Left and Right Ventricular Mass and Volumes on Cardiac MRI - Image analysis for left and right ventricular volumes and mass will be performed offline on the short-axis cine stack with commercially available software (cvi/cmr42, Circle Cardiovascular Imaging Inc., Calgary, Canada) as previously described [21, 41]. Left and right ventricular short-axis epicardial and endocardial borders will be manually contoured at end diastole and end systole to allow automated calculation of left and right ventricular mass and volumes. Mass represents the following: (end-diastolic epicardial−endocardial volume) × 1.05. Stroke volume is end-diastolic volume minus end-systolic volume, and ejection fraction is given by the following: (stroke volume/end-diastolic volume) × 100%. Left ventricular wall thickness will be measured on the midventricular short-axis slice at end diastole, and internal and external left ventricular cavity diameters will be measured on the midventricular short-axis slice at end diastole between the septum and inferolateral wall. Left ventricular length will be measured at end diastole on the horizontal long-axis cine between the left ventricular apex and middle of the mitral annulus. Left ventricular relative wall thickness will be calculated as follows: (2 × inferior wall thickness)/end-diastolic diameter. Right ventricular length will be measured at end diastole on the horizontal long axis cine by drawing a line along the tricuspid valve annulus and measuring the length from the right ventricular apex to the middle of the tricuspid valve annulus. Right ventricular diameter measurements will be made on the horizontal long axis cine at end diastole within the basal third of the right ventricle below the tricuspid valve and at the level of the right ventricular papillary muscles.

Assessment of Ventricular Myocardial Deformation on Cardiac MRI - In addition to gross volumetric measures of systolic left and right ventricular function (ejection fraction and stroke volume), systolic and diastolic function and cardiac rotational movement will be measured on the basis of myocardial deformation parameters assessed with commercially available software (cvi/cmr42, Circle Cardiovascular Imaging Inc., Calgary, Canada). The left ventricular endocardial borders of the steady-state free-precession horizontal long-axis, vertical long-axis and left ventricular outflow tract cines and basal, mid, and apical left ventricular short-axis cines will be manually contoured on the end-diastolic frame. Right ventricular myocardial deformation will be assessed on the horizontal long axis cine only. The software tracks the motion of related features adjacent to the endocardial line such as the cavity-tissue boundary or individual tissue patterns over the cardiac cycle to produce endocardial strain parameters [42].

Cardiac Computational Atlas Formation – Creation of a cardiac statistical atlas of all cardiovascular magnetic resonance images will be undertaken. The end-diastolic frame from the DICOM file for each slice of the right and left ventricular short axis cine stack that includes the manually contoured endocardial and epicardial contours drawn using cvi42/cmr42 will be retrieved and rebuilt into a single DICOM file in Matlab. The file will then be converted into a binary segmentation image representing the right and left ventricles, and a mesh fitted to this myocardial anatomy, achieving subvoxel accuracy. The right and left ventricles for each subject will then be described with a mesh definied by a set of nodal variables. Principal and linear component analysis will be undertaken to identify the key modes of variation of the shape [21].

#### Aortic MRI

Aortic distensibility will be assessed to gain insight into aortic stiffness. A transverse image obtained at the level of the right pulmonary artery planned off an oblique sagittal view of the aorta allows for the cross-sectional assessment of the ascending aorta, proximal descending aorta and pulmonary artery with its branches [43, 44]. Maximum and minimum aortic cross-sectional areas over the cardiac cycle will be determined using semi-automated edge detection algorithms developed using Matlab software (Mathworks Inc.) and distensibility will be calculated as the relative change in area divided by the pulse pressure: (aortic distensibility = {[(maximum area of the aorta – minimum area of the aorta)/(minimum area of the aorta)]/aortic pulse pressure} × 10^3^). A surrogate measure of aortic blood pressure using sphygmomanometer-derived indices will be acquired with a blood pressure cuff around the right arm (Vicorder, Skidmore Medical, Taunton, UK) during the MRI scan for the calculation of aortic distensibility.

#### Hepatic and abdominal MRI

Transverse abdominal liver T_1_ and T_2_* MR maps and DIXON liver images are acquired for the estimation of extracellular fluid, liver iron, and liver steatosis respectively. T_1_ relaxation time increases with increases in extracellular fluid, such as in fibrosis and inflammation. However, the presence of iron, which can be accurately measured from T_2_* maps, has an opposing effect on the T_1_. An algorithm has been created that allows for the bias introduced by elevated iron to be removed from the T_1_ measurements, yielding the cT_1_. LiverMultiScan (Perspectum Diagnostics, Oxford, United Kingdom) is a software product specifically developed to measure cT_1_ from T_1_ and T_2_* maps. For TEPHRA, the LiverMultiScan will be used to analyse cT_1_ in at least a single, operator-defined region of the liver away from vascular and biliary structures. DIXON images will be used to quantify proton-density fat fraction to assess liver steatosis. Transverse abdominal multi-slice T1-weighted TSE images at the level of the 5th lumbar vertebra will be acquired to measure visceral and subcutaneous adipose tissue.

### Sample size

The rationale for the number of participants required to address the primary outcome is as follows. Our systematic review and meta-analysis of randomised control trials [14] identified an average decrease of 5.4 mmHg in systolic blood pressure (SBP) post-intervention in adults with prehypertension (mean systolic blood pressure at baseline 126 mmHg) [14]. The post intervention standard deviation of change was 8.7 mmHg. Our available cross-sectional pilot data suggests that change will be higher in the preterm born participants and participants exposed to maternal hypertension during pregnancy. To calculate the sample size we have been conservative and used an estimated change of 5 mmHg following 16 weeks of intervention. We have used a standard deviation (SD) of 11.3 from the pooled SD for ambulatory systolic blood pressure from our cross-sectional pilot data. To observe a treatment effect on systolic blood pressure of 5 mmHg, powered to 80% (*p* = 0.05) requires a total sample size of 164 participants. The SD deviation for the pooled ambulatory diastolic blood pressure is 8.3 mmHg. To observe a treatment effect on diastolic blood pressure of 5 mmHg, powered to 80% (p = 0.05) requires a total sample size of 114 participants. The power calculations for systolic blood pressure are used to determine the final sample size, with adjustment to 200 participants to allow for 18% attrition. To ensure primary objectives for the study are answered, the study team may recruit additional participants to replace those who drop out and withdraw from the study.

This sample size will provide 80% power and 5% significance to analyse the proposed secondary outcomes.

### Randomisation

Participants meeting the study inclusion criteria will be randomly allocated 1:1 to the two intervention arms. This will be undertaken using Sealed Envelope™ (https://www.sealedenvelope.com/), a computerised randomisation program. A minimisation algorithm (with a random element of 80%) is used to ensure balanced allocation across the two groups for key prognostic factors: gender, age and gestational age of participants. The strata for each prognostic factor are as follows:

- Gender: male/female;
- Age: < 24 years old, 24–29 years old, 30–35 years old;
- Gestational age: ≤32 weeks, 32–37 weeks, > 37 weeks.

Allocation is concealed following the completion of baseline study measures and only designated trial team members monitoring potential exercise injuries and adverse events and the trial statistician will be made aware of each participant’s allocation. Outcome assessors will be blinded to reduce potential bias. Due to the nature of the intervention, participants will not be blinded.

An emergency randomisation schedule has been prepared in advance and stored for use when the online system in unavailable. The randomisation list was created using simple block randomisation using variable block size.

### Study intervention

Participants in the intervention arm will complete 16 weeks of structured aerobic exercise training, with a target of 3 training sessions per week, duration 60 min, at exercise intensity of 60–80% aerobic exercise capacity. Exercise intensity of 60–80% maximal aerobic capacity defined using heart rate response during peak cardiopulmonary exercise testing at baseline. Heart rate monitors will be used to maintain training intensity. The intervention replicates similar strategies identified during systematic review of randomised control trials delivering exercise intervention for blood pressure reduction [14]. The minimum dose to start effecting positive changes in cardiovascular fitness is estimated to be 40 min of moderate intensity sessions three times per week. The study team will monitor participants’ compliance and progress during the structured aerobic exercise training. The study team will use motivational coaching strategies to encourage participants to maintain compliance with the training program. In addition to structured aerobic exercise training, participants will be provided with a wrist worn heart rate and accelerometer based activity monitor (Fitbit Charge HR) to facilitate physical activity self-monitoring and maintenance of heart rate targets. Participants will be consented to allow investigators access to the data collected from wear of the Fitbit. In addition, participants will be sign-posted to educational materials produced by the British Heart Foundation explaining blood pressure, blood pressure prevention and recommended lifestyle behaviours to maintain heart health.

On completion of the structure aerobic exercise training participants will be encouraged to continue to use their activity monitor and maintain physical activity goals until the final study visit at 52 weeks. The use of the activity monitors will support the three most effective behaviour change strategies to promote increased physical activity, including self-monitoring, goal setting and regular feedback [45, 46]. To encourage continued regular, self-directed physical activity, participants will be provided a motivational coaching session on completion of the structured 16 week training program. A study team member trained in motivational interview techniques will provide the motivational coaching. Participants will be asked to reflect on their experience of physical activity, their experience of participating in the structured aerobic exercise training, will be encouraged to set goals and rate their confidence and motivations to achieve physical activity goals, participants will be encouraged to discuss strategies to maintaining physical activity participation.

### Comparison group

Participants in the control group will be sign-posted to educational materials produced by the British Heart Foundation explaining blood pressure, blood pressure prevention and recommended lifestyle behaviours to maintain heart health. Participants will be asked to continue with their regular physical activity routine with no change.

### Withdrawals

Each participant has the right to withdraw from the trial at any time. In addition, the Chief Investigator may discontinue a participant from the study at any time if he considers it necessary for any reason including:

- Pregnancy;
- Ineligibility (either arising during the study or retrospectively having been overlooked at screening);
- Significant deviation from the study protocol;
- Withdrawal of consent;
- Loss to follow up;
- Contraindication to exercise participation.

If participants withdraw from the study, the study investigators will ask the participants if they can make use of the information that has been collected up to the time of withdrawal to facilitate intention to treat analysis. Participants may withdraw consent for any use of their samples or data at any time. If this is the case, the trial team will destroy any identifiable samples or information held about the participant. Participants do not need to provide any reason for withdrawal; should they freely offer a reason it will be recorded in the case report form (CRF). The research team may recruit additional participants to replace participants who have withdrawn.

### Participant confidentiality

The study staff will ensure that the participants’ anonymity is maintained. Only the participant’s ID number on the CRF and in the electronic database will identify the participants. All documents will be stored securely and only accessible by study staff and authorised personnel. The study will comply with the General Data Protection Regulation and the United Kingdom Data Protection Act, which requires data to be anonymised as soon as it is practical to do so.

### Data management

Direct access to the data will be granted to authorised representatives from the Sponsor or host institution for monitoring and/or audit of the study to ensure compliance with regulations. Data management will commence with formation of a study database. Only a unique participant number on any electronic database will identify the participants. All documents will be stored securely and only accessible by study staff and authorised personnel. Electronic data will be encrypted and password protected, and stored on departmental computers. Access will be granted only to members of the study team and authorised personnel. The study will comply with the United Kingdom Data Protection Act, which requires data to be anonymised as soon as it is practical to do so. Only one electronic study document will contain identifiable information; this is the enrolment log combined with the “code break” which links the unique participant number with participant name and contact details. This will be encrypted, stored on a high security server and only accessible by study staff and authorised personnel. Study duration of 3 years is anticipated and study data will be kept for 7 years following completion of the study.

### Safety considerations

Overall this is a low risk study. Exercise training is safe and the established global benefits to health and well-being outweigh negative complications and risks. However, risk of injury, medical complications and serious adverse events are reported in association with exercise participation. Muscle and joint soreness and minor musculoskeletal complaints are common adverse effects of exercise training, however the risk of a serious adverse event including sudden cardiac death or non-fatal cardiac event is reported as below 0.01 per 10,000 h of participation. The detailed screening and cardiovascular assessment prior to randomization will identify any participants at potential cardiovascular risk associated with exercise participation, if there is any concerns regards an individual’s participation they will be referred for appropriate clinical review and excluded from the trial. Should an participant experience or report any adverse events or symptoms at any time during the study period they will be advised on how to manage these symptoms and will be advised to seek further medical evaluation if required via their primary health care practitioner or local emergency medicine department.

### Adverse events

All adverse events occurring during the trial or until completion of the final study visit that are observed by the Chief Investigator or reported by the participant will be recorded on the study case report form (CRF), whether or not attributed to the exercise intervention.

The following information will be recorded: description, date of onset and end date, severity, and assessment of relatedness to exercise intervention, other suspect drug or device and action taken. Follow-up information should be provided as necessary.

Adverse events considered related to the trial as judged by a medically qualified investigator or the Sponsor will be followed either until resolution, or the event is considered stable.

It will be left to the Investigator’s clinical judgment to decide whether or not an AE is of sufficient severity to require the participant’s removal from the study. A participant may also voluntarily withdraw from intervention due to what he or she perceives as an intolerable adverse event. If either of these occurs, the participant must undergo an end of trial assessment and be given appropriate care under medical supervision until symptoms cease, or the condition becomes stable.

Safety reporting will be from baseline to the 12-month follow up visit. The participant using the trial telephone number or email may also report adverse events. Participants will be directly asked about adverse events at each study visit.

Serious adverse events (SAE) occurring to a participant will be reported to the research ethics that gave a favourable opinion of the study where in the opinion of the Chief Investigator the event was ‘related’ (resulted from administration of any of the research procedures) and ‘unexpected’ in relation to those procedures. Reports of related and unexpected serious adverse events will be submitted within 15 working days of the Chief Investigator becoming aware of the event, using a human research authority serious adverse event form.

### Data analysis

The analysis will be carried out on the basis of intention-to-treat. This is, after randomisation, participants will be analysed according to their allocated intervention group irrespective of what they actually receive during the specified period.

Patient demographic characteristics and other baseline information will be summarised by intervention group. Numbers (with percentages) for binary and categorical variables and mean (standard deviation), or median (interquartile or full range) for continuous variables will be presented. Normality of variables will be assessed by visual assessment of the normality curves and the Shapiro-Wilk test. Methods such as the Student T-test will assess for differences between the groups. The analysis of the primary outcome will be assessed using analysis of covariance (ANCOVA) adjusting for baseline values and minimisation factors used in the randomisation process. Results will be presented as adjusted mean difference in change in ambulatory blood pressure between randomised groups at 16 weeks with 95% confidence intervals and associated two-sided *p* value. No imputation will be carried out and analysis will be performed on all available data. Sensitivity analyses will be performed on the per-protocol populations. The definitions of these will be detailed in the statistical analysis plan.

The analysis of secondary outcomes will also be done using analysis of covariance (ANCOVA) to establish a statistical model to examine the effect of cardiac structure, vascular function and lifestyle behaviours on exercise capacity. Outcomes measured on more than one occasion will be analysed using a mixed effects model. If the model assumptions are not met and evidence of departure from Normality is observed, transformations of the data will be employed or non-parametric tests will be carried out. Statistical analysis will be carried out using STATA, SPSS or R statistical software. A detailed statistical analysis plan will be written with our statistical team and will be completed before receipt of the data.

### Trial oversight

A Trial Steering Committee (TSC) has been formed to oversee the conduct of the trial. The TSC consists of the Chief Investigator, independent clinical experts who specialise in areas relevant to the trial (primary care hypertension, sports medicine and rheumatology, and physiotherapy), the trial statistician and an independent statistician. The TSC meets approximately every six months, either in person or via teleconference. A data and safety monitoring committee (DSMC) has also been formed with the appropriate specialties to provide insight and oversight to the trial progression and management. Rates of recruitment, trial compliance, measurement of the primary outcome and study attrition are reviewed biannually by the independent members, including an independent statistician, of this committee. The data monitoring committee meets approximately every six months, and shortly prior to TSC meetings when possible.

### Dissemination policy

An authorship plan has been drafted reflecting the described primary and secondary objectives for the study. A full manuscript detailing the primary outcomes will be submitted within 12 months of completing the trial with secondary outcomes reported within 24 months of completing the trial. Completion of study defined as full participant enrolment and data collection at 52 weeks follow-up being completed.

## Discussion

The TEPHRA trial is the first study with the primary aim of determining the effects of structured aerobic exercise and physical activity self-monitoring intervention on ambulatory blood pressure in young adults [14]. Furthermore, it is the first study to explore the effects of exercise intervention with reference to novel cardiovascular risk factors such as preterm birth [14, 18]. The association between maintaining high cardiovascular fitness and physical activity and the benefit for cardiovascular and cerebrovascular health are well documented [47–50]. However, there is a paucity of trial evidence supporting exercise and physical activity intervention as an effective treatment option with sustained benefits in young adult populations [14]. Despite this lack of evidence lifestyle intervention is recommended as the primary intervention in young adults with hypertension [23]. In addition, there is limited understanding, with minimal prospective evidence in a trial setting, demonstrating the multisystem physiological adaptations to exercise which may mediate the benefits on future cardiovascular and cerebrovascular risk.

The results from the TEPHRA trial will provide preliminary evidence to support whether or not exercise and physical activity intervention should be primary treatment options for management of blood pressure. It will also provide evidence exploring potential heterogeneity in response to exercise intervention with reference to novel cardiovascular risk factors and baseline phenotypes such as those associated with preterm birth. The detailed cardiovascular and cerebrovascular phenotyping, using multimodal imaging, resting and stress measures of cardiovascular function will facilitate insights into disease mechanisms and phenotypic traits, which may demonstrate plasticity and remodelling, and may identify target mechanisms and interim outcomes for future intervention. The results will expand the available evidence-base for the management of blood pressure in young adult populations and inform the future direction of research targeting early cardiovascular risk reduction.

### Trial status

Recruitment is on-going.

## Abbreviations

ABP: Ambulatory Blood Pressure
AE: Adverse Event
ANCOVA: Analysis of Covariance
ASL: Arterial Spin Labelling
AVR: Arteriolar-to-Venular-Ratio
BHF: British Heart Foundation
BIANCA: Brain Intensity AbNormality Classification Algorithm
BMI: Body Mass Index
CI: Chief Investigator
CPET: Cardiopulmonary Exercise Testing
CRAE: Central Retinal Arteriolar Equivalents
CRF: Case Report Form
CRVE: Central Retinal Venular Equivalents
CSD: Constrained Spherical Deconvolution
DALYs: Disability-Adjusted Life Years
DBP: Diastolic Blood Pressure
DSMC: Data and Safety Monitoring Committee
DTI: Diffusion Tensor Imaging
DWI: Diffusion Weighted Imaging
ECFC: Endothelial Colony-Forming Cells
ECG: Electrocardiogram
EPI: Echo-Planar-Imaging
FA: Fractional Anisotropy
FLAIR: Fluid Attenuated Inversion Recovery
FOV: Field-of-View
FSL: FMRIB Software Library
GP: General Practice
ID: Identity
MIST: Multimodal Image Segmentation Tool
MRA: Magnetic Resonance Arterogram
MRI: Magnetic Resonance Imaging
NHS HRA: National Health Service Health Research Authority
NICE: National Institute for Health and Care Excellence
NIHR: National Institute for Health Research
PMBCs: Peripheral Blood Mononuclear Cells
REC: Research Ethics Committee
RPAQ: Recreational Physical Activity Questionnaire
SAE: Serious Adverse Event
SBP: Systolic Blood Pressure
SD: Standard Deviation
TE: Echo Time
TEPHRA: Trial of Exercise to Prevent HypeRtension in young Adults
TOF: Time of Flight
TR: Repetition Time
TSC: Trial Steering Committee
TSE: Turbo Spin Echo
UK: United Kingdom
WMH: White matter hyperintensity
